# Pruritus Ani

**Published:** 1895-08

**Authors:** 


PRURITUS ANI.—This rather common malady, so exceedingly disagreeable to the patient, is almost equally annoying to the physician on account of the obstinacy with which it resists therapeutic measures. Many varied causes may combine to produce the disease. It is sometimes due to hemorrhoids; is often the result of eczema of the skin about the verge of the anus; and sometimes is a purely nervous affection. Belladonna, opium, cocaine, antipyrin, hot cloths, and hot preparations of vinegar are useful. Berger recommends, as a very successful measure, cotton compresses wet in a concentrated solution of chloride of lime and passed into the anus, to be retained a few minutes if the patient does not complain too much of the burning. The external parts are bathed with the same solution and allowed to dry. This method rapidly relieves the unnatural itching, also cures the surrounding eczematous eruptions. The concentration of the solution must be graduated to suit the tolerance of the patient.—*Dietetic and Hygienic Gazette.*

